# Powassan Meningoencephalitis, New York, New York, USA

**DOI:** 10.3201/eid1909.121846

**Published:** 2013-09

**Authors:** Simon Sung, Alysse G. Wurcel, Susan Whittier, Karen Kulas, Laura D. Kramer, Robin Flam, James Kirkland Roberts, Simon Tsiouris

**Affiliations:** Columbia University, New York City, New York, USA (S. Sung, A.G. Wurcel, S. Whittier, R. Flam, J.K. Roberts, S. Tsiouris);; Tufts Medical Center, Boston, Massachusetts, USA (A.G. Wurcel);; New York State Department of Health, Albany, New York, USA (K. Kulas, L.D. Kramer);; Mid Hudson Medical Group, Hopewell Junction, New York, USA (R. Flam)

**Keywords:** Powassan virus, encephalitis, meningoencephalitis, meningitis, arbovirus, flavivirius, tick-borne disease, viruses, vector-borne infections, New York

## Abstract

Disease caused by Powassan virus (POWV), a tick-borne flavivirus, ranges from asymptomatic to severe neurologic compromise and death. Two cases of POWV meningoencephalitis in New York, USA, highlight diagnostic techniques, neurologic outcomes, and the effect of POWV on communities to which it is endemic.

Powassan virus (POWV), a rare neuroinvasive arbovirus, was first described in 1958 (*1*,*2*). POWV has been isolated from *Ixodes* ticks; implicated hosts include woodchucks, red squirrels, chipmunks, groundhogs, and white-footed mice (*1*–*4*). Symptoms of infection vary from mild myalgia to acute flaccid paralysis and neurologic involvement. In the United States, POWV has been reported in northeastern and north-central states, and incidence is increasing (*3*,*4*). We describe the clinical characteristics and outcomes of 2 patients with POWV in New York, New York, USA. 

## Case Reports

### Case 1

In mid-February 2009, a 22-year-old man with a remote history of Lyme disease (LD) was transferred to Columbia University New York Presbyterian Hospital (CUNYPH) after an extensive work-up for aseptic meningitis. On December 20, 2008, he had flown home to eastern Long Island, New York, from Colorado. Sore throat and influenzaa-like symptoms developed, and he sought care from a local physician in early January. Rapid strep test result was negative, but oral cephalosporin was prescribed. His symptoms improved, and he returned to Colorado. Approximately 2 weeks later, symptoms recurred, along with fever, eye pain, lateral gaze palsy, ataxia, dysarthria, stomach pain, and neck stiffness. After visiting the student health center, he was admitted to a local hospital in early February. Results of blood tests were within normal limits; cerebrospinal fluid (CSF) contained 212 leukocyte/μL (reference 0–5 cells/μL) (95% lymphocytes), 0 erythrocytes/μL (reference 0/μL), protein 55 mg/dL (reference 15–60 mg/dL), and glucose 60 mg/dL (reference 50–80 mg/dL). He received intravenous ceftriaxone and acyclovir, improved slightly, and was discharged after 1 week. He returned to New York and was admitted to a local hospital 1 week after discharge with persistent neurologic signs. Repeat CSF analyses showed 59 leukocytes/μL (100% lymphocytes); given concern about LD, he was placed on ceftriaxone until LD serologic results were negative. All other PCRs and serologic work-ups for infectious agents were negative (Technical Appendix, Table).

Given limited improvement, he was transferred to CUNYPH for reevaluation. On arrival, neurologic examination revealed decreased alertness, dysarthria, and ataxia. T2-weighted magnetic resonance imaging of the brain showed bilateral caudate and basal ganglia hyperintensities consistent with viral encephalitis (Technical Appendix, Figure 1, panels A, B). He reported raising chickens, frequently drinking unpasteurized milk, and hiking in a wooded area in Long Island during holiday break, and his college roommate had a recent “mono-like” illness. Results of serologic tests on serum and CSF collected in mid-February showed polyvalent antibodies to POWV (Table). POWV meningoencephalitis was confirmed with >4-fold change in POWV plaque reduction neutralizing test (PRNT) titers in paired serum samples: February titer, 20; March titer, 320. At discharge in late February, symptoms improved with mild residual tremor, but dysarthria persisted. At 1-month follow-up, persistent dysarthria remained, but other symptoms had resolved completely.

**Table:** Results of initial arboviral serologic testing and POWV RT-PCR on CSF from 2 patients, performed at Wadsworth Center, New York State Department of Health, New York, USA*

Test	Test result, month	
	Patient 1, 2009	Patient 2, 2012
West Nile virus IgM ELISA	Nonreactive, Feb	Nonreactive, May
Eastern equine encephalitis virus IgG IFA	<16, Feb	<16, May
Western equine encephalitis virus IgG IFA	<16, Feb	<16, May
California serogroup IgG IFA	<16, Feb	<16, May
St. Louis encephalitis virus IgG IFA	<16, Feb	<16, May
rPOW-E polyvalent MIA† (titer)	Reactive, Feb	Reactive, May
Powassan PRNT (titer)	+ (20), Feb	+ (280), early May
	+ (320), Mar	+ (320), late May
		+ (160), early Jun
		+ (10), late Jun
Powassan RT-PCR CSF	Not done	–

*POWV, Powassan virus; RT-PCR, reverse transcription PCR; CSF, cerebrospinal fluid; IFA, immunofluorescent assay; MIA, microsphere immunoassay; PRNT, plaque reduction neutralizing test;; +, positive; –, negative. †A fluorescent MIA technique using recombinant POWV envelope glycoprotein antigen to detect IgG, IgA, and IgM.

### Case 2

In early May 2012, a 34-year-old man with a history of genital herpesvirus infection was admitted to a local hospital with lower extremity weakness and altered mental status of unclear etiology. In late April 2012, headache, fever, chills, and bilateral ankle pain had developed. Despite treatment with oral doxycycline for presumptive LD, symptoms persisted; development of bilateral leg weakness, confusion, and diplopia prompted evaluation at the local hospital 1 week after symptom onset. He was afebrile and hemodynamically stable; physical examination revealed decreased alertness, bilateral proximal leg weakness, and absence of neck rigidity. Further questioning revealed a history of 2 transient rashes (1 each on trunk and arm) 1 week before symptom onset, but they were not visible on physical examination. CSF showed 145 leukocytes/μL (100% lymphocytes), 35 erythrocytes/μL, protein 142 mg/dL, and glucose 39 mg/dL. He received intravenous vancomycin, ceftriaxone, and acyclovir. T2-weighted magnetic resonance imaging of the brain showed hyperintensities in bilateral temporal lobes (Technical Appendix, Figure 2). Ceftriaxone was discontinued after the LD test result was negative. Results of all other infectious disease tests were negative (Technical Appendix, Table).

Because of worsening symptoms, the patient was transferred to CUNYPH 3 days later. Repeat CSF analysis showed 78 leukocytes/μL with lymphocyte predominance. The patient resided in rural New York, had a remote history of tick bites, and had recently disposed of a bird nest. His wife worked as a daycare assistant, and he had 2 healthy children <5 years of age.

Serum was sent to the New York State Department of Health, and testing for POWV was requested after the family reported that, 2 years earlier, a neighborhood child 2 years was infected with POWV. POWV reverse transcription PCR in CSF sent from his previous hospital was negative; however, polyvalent antibody to POWV was detected in serum collected in early May. POWV infection was confirmed by >4-fold change in serum POWV PRNT titers in paired serum samples: early May, 1,280; late May, 320; early June, 160; and late June, 10. Serologic results for other arboviral diseases were negative (Table). On day 10 of hospitalization, his mental status returned to baseline, and he began to walk with a rolling walker. He was discharged on day 11 to an acute-care rehabilitation facility. Two weeks after discharge, the patient reported major improvement in leg strength and took a few steps unassisted. His symptoms relapsed 1 month after hospitalization, prompting admission for a trial of intravenous steroid therapy (1,000 mg methylprednisolone for 3 days). Repeat work-up for encephalitis was negative for all viral, bacterial, and fungal diseases, except positive for POWV antibodies. His symptoms improved, and he walked assisted by a rolling walker. Six months after the initial admission, he had improved substantially with outpatient physical therapy; after 10 months, he returned to work despite residual leg weakness .

## Conclusions

In 2011, the Centers for Disease Control and Prevention confirmed 16 cases of POWV infection—a substantial increase from previous years but most likely an underestimate because underdiagnosis is probably common (*4*–*6*). New York State alone reported 12 POWV neuroinvasive cases during 2001–2010 (*5*). Although most cases reported in the literature occurred during June–September, tick feeding and transmission of POWV can extend into the milder periods of spring, autumn, and even winter. The clinical characteristics, course of disease, and residual neurologic deficits vary (*3*,*7*–*11*).

As with other arboviral infections, POWV diagnosis is complicated and requires knowledge of and access to diagnostic tests (*12*). Physicians in areas to which POWV is endemic should be aware of POWV and request serologic testing on serum or CSF samples from the state or other laboratories capable of performing POWV serologic tests. CSF PCR for POWV may be negative, especially if patients seek care later in their clinical course, and PRNT may be useful, especially when clinical suspicion is high. Although increasing PRNT titers suggest acute infection, we cannot determine the significance of the change in titers over time. A cohort study with long-term follow-up of POWV-infected patients with careful documentation of serology and radiologic images would help with prognostication; identify risk factors for more severe disease; and measure the effect of interventions, such as corticosteroid therapy.

Because no vaccines or effective antiviral agents exist for POWV, physicians in disease-endemic areas should advise their patients to take precautions to prevent tick bites, including wearing light-colored clothes with pants tucked into socks, using tick repellent, and carefully inspecting themselves and their pets for ticks after spending time outdoors (*13*). In addition, physicians should be aware of the existence, epidemiology, prognosis, diagnosis, and complications of POWV infection because this disease is likely to increase in areas to which it is endemic.

Technical Appendix Results of laboratory diagnostic tests for patients 1 and 2 and magnetic resonance imaging results of bilateral basal ganglia and caudate hyperintensities on fluid attenuated inversion recovery/T2-weighted sequence in patient 1; and T2/magnetic resonance imaging results of bilateral temporal hyperintensities on fluid attenuated inversion recovery/T2-weighted sequence in patient 2.
