# Supplementary material for: Powassan Meningoencephalitis, New York, New York, USA
Source: Emerg Infect Dis. 2013 Sep;19(9):1549–51. doi: 10.3201/eid1909.121846 (PMC3810908; doi:10.3201/eid1909.121846)

Publisher: CDC; Journal: Emerging Infectious Diseases
Article Type: Dispatch; Volume: 19; Issue: 9; Year: 2013; Article ID: 12-1846
DOI: 10.3201/eid1909.121846; TOC Head: Dispatch
Article DOI: http://dx.doi.org/10.3201/eid1909.121846

# Powassan Meningoencephalitis, New York City, USA

## Technical Appendix

Technical Appendix Table. Results of laboratory diagnostic tests on CSF and serum for 2 patients with Powassan virus infection, New York City, New York, USA"

| Test | Patient 1 | Patient 2 |
|---|---|---|
| CSF PCR | – | – |
| Adenovirus | – | ND |
| Enterovirus | – | – |
| Ehrlichichia | – | – |
| Epstein-Barr virus | – | – |
| Herpes simplex virus 1 and 2 | – | – |
| Human herpesvirus-6 | | |
| Varicella-zoster virus | – | – |
| CSF culture | – | – |
| Bacteria | – | – |
| Fungi | – | – |
| *Mycobacterium tuberculosis* | | |
| CSF cryptococcus antigen | ND | – |
| Serum antibody test | – | – |
| *Anaplasma phagocytophilium* | – | ND |
| HIV | – | – |
| *Babesia* IgG, IgM | ND | – |
| Cytomegalovirus IgG | | |
| *Ehrlichia chaffeensis* IgG | | |

*CSF, cerebrospinal fluid;–, negative; ND, not done; + positive.

Publisher: CDC; Journal: Emerging Infectious Diseases
Article Type: Dispatch; Volume: 19; Issue: 9; Year: 2013; Article ID: 12-1846
DOI: 10.3201/eid1909.121846; TOC Head: Dispatch

Technical Appendix Figure 1. Patient 1: Magnetic resonance imaging FLAIR/T2 showing (A) bilateral basal ganglia and (B) caudate hyperintensities. FLAIR, fluid attenuated inversion recovery.

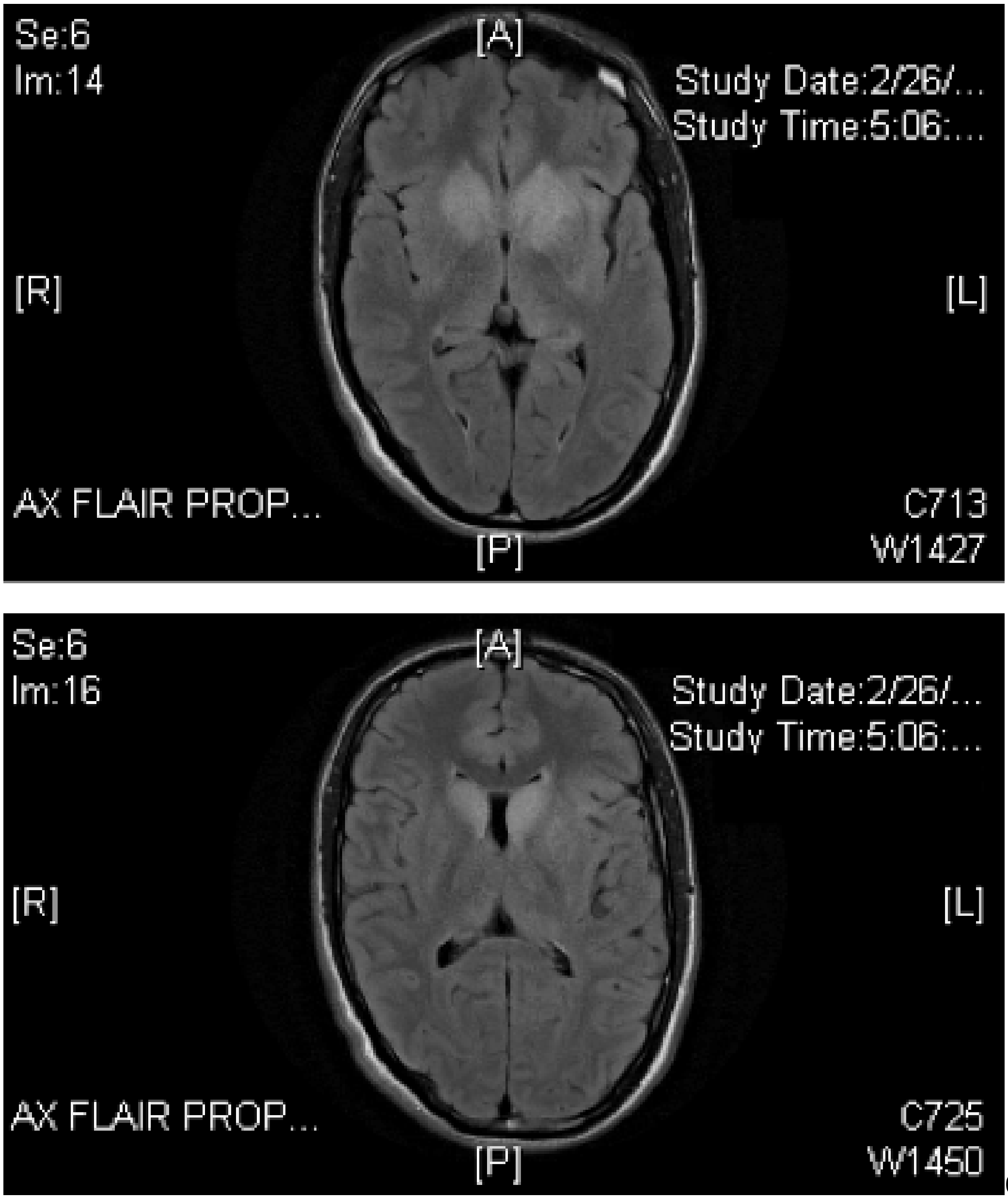

0

Publisher: CDC; Journal: Emerging Infectious Diseases
Article Type: Dispatch; Volume: 19; Issue: 9; Year: 2013; Article ID: 12-1846
DOI: 10.3201/eid1909.121846; TOC Head: Dispatch

Technical Appendix Figure 2. Patient 2: Magnetic resonance imaging FLAIR/T2 showing bilateral temporal hyperintensities. FLAIR, fluid attenuated inversion recovery.

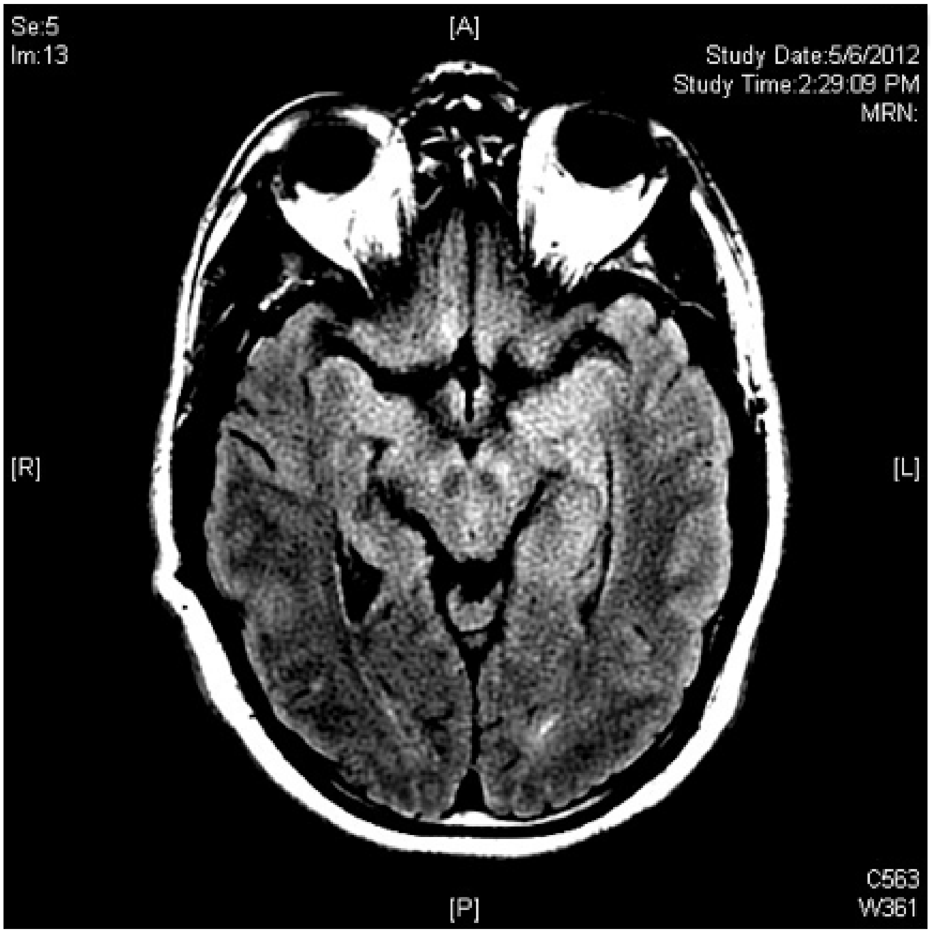

Supplement: Technical Appendix — Results of laboratory diagnostic tests for patients 1 and 2 and magnetic resonance imaging results of bilateral basal ganglia and caudate hyperintensities on fluid attenuated inversion recovery/T2-weighted sequence in patient 1; and T2/magnetic resonance imaging results of bilateral temporal hyperintensities on fluid attenuated inversion recovery/T2-weighted sequence in patient 2. [file 12-1846-Techapp-s1.pdf]
